# Effects of Successive Rotation Regimes on Carbon Stocks in Eucalyptus Plantations in Subtropical China Measured over a Full Rotation

**DOI:** 10.1371/journal.pone.0132858

**Published:** 2015-07-17

**Authors:** Xiaoqiong Li, Duo Ye, Hongwen Liang, Hongguang Zhu, Lin Qin, Yuling Zhu, Yuanguang Wen

**Affiliations:** 1 State Key Laboratory for Conservation and Utilization of Subtropical Agro-bioresources, College of Forestry, Guangxi University, Nanning, Guangxi, China; 2 Guangxi Colleges and Universities Key Laboratory of Forestry Science and Engineering, College of Forestry, Guangxi University, Nanning, Guangxi, China; Tennessee State University, UNITED STATES

## Abstract

Plantations play an important role in carbon sequestration and the global carbon cycle. However, there is a dilemma in that most plantations are managed on short rotations, and the carbon sequestration capacities of these short-rotation plantations remain understudied. Eucalyptus has been widely planted in the tropics and subtropics due to its rapid growth, high adaptability, and large economic return. Eucalyptus plantations are primarily planted in successive rotations with a short rotation length of 6~8 years. In order to estimate the carbon-stock potential of eucalyptus plantations over successive rotations, we chose a first rotation (FR) and a second rotation (SR) stand and monitored the carbon stock dynamics over a full rotation from 1998 to 2005. Our results showed that carbon stock in eucalyptus trees (TC) did not significantly differ between rotations, while understory vegetation (UC) and soil organic matter (SOC) stored less carbon in the SR (1.01 vs. 2.76 Mg.ha^-1^ and 70.68 vs. 81.08 Mg. ha^-1^, respectively) and forest floor carbon (FFC) conversely stored more (2.80 vs. 2.34 Mg. ha^-1^). The lower UC and SOC stocks in the SR stand resulted in 1.13 times lower overall ecosystem carbon stock. Mineral soils and overstory trees were the two dominant carbon pools in eucalyptus plantations, accounting for 73.77%~75.06% and 20.50%~22.39%, respectively, of the ecosystem carbon pool. However, the relative contribution (to the ecosystem pool) of FFC stocks increased 1.38 times and that of UC decreased 2.30 times in the SR versus FR stand. These carbon pool changes over successive rotations were attributed to intensive successive rotation regimes of eucalyptus plantations. Our eight year study suggests that for the sustainable development of short-rotation plantations, a sound silvicultural strategy is required to achieve the best combination of high wood yield and carbon stock potential.

## Introduction

Forests represent an important carbon sink, thus playing a key role in the global carbon cycle [1, 2]. As forested land is able to store more carbon per unit area than any other terrestrial ecosystem, forestation may act as an effective measure in mitigating global climate warming [3, 4]. Understanding the role of forest plantations as carbon reservoirs is crucial, in order to improve predictions of how land use change may impact the global carbon cycle. Recent studies have revealed an increase in carbon sequestration in plantations over the last several decades [5, 6]. However, there is a dilemma in that most of plantations are managed with a rotation period of less than 20 years, and the carbon sequestration capacity of such short-rotation forests is poorly investigated [7].

The carbon sequestration capacity of plantations may be influenced by many other factors, such as climate [8], forest management strategy [9], frequency of disturbance [10], functional groups present [11], soil conditions [12], tree species [13], and stand age [14]. Multi-species plantations are likely to demonstrate a greater productivity and can enhance soil carbon sequestration [13, 15, 16]. Many studies also reported that the use of different site preparation techniques can greatly affect carbon stock potential in plantations [10, 15]. Higher levels of disturbance, including site preparation, fertilization, weeding, thinning and harvesting, have been shown to influence the decomposition rate of detritus and soil carbon dynamics [17–19]. Carbon stock dynamics in short-rotation plantations have been previously characterized [3, 15, 16, 20], but results were inconsistent, perhaps due to the short-term (often single rotation) nature of the data. Further study and longer-term evaluation of carbon sequestration potentials, as well as other ecosystem services provided by plantations, are required.

Eucalyptus trees (e.g., *E*. *urophylla* × *E*. *grandis*, *E*. *urophylla*, and *E*. *grandis*) are widely planted in the tropics and subtropics, not only in subtropical China but throughout the world, as they grow quickly and produce commercially desirable goods [5, 21]. The earliest plantings of eucalyptus in China can be dated back to 120 years ago [22]. With recent large-scale afforestation and reforestation efforts, eucalyptus plantations in China have expanded rapidly, covering 4.6 million hectares in 2014. These afforested areas are primarily managed as successive short-rotation (around 6~8 years) plantations in subtropical China [23], despite frequent reports from forest managers and scientists of yield declines, degradation of soil fertility and reductions in biodiversity over successive eucalyptus plantation rotations [5, 23–25]. The effect of successive rotations on the carbon stock capacity of eucalyptus plantations remains poorly understood, however.

Therefore, our objective was to evaluate the carbon-stock capacity of eucalyptus plantations in two successive rotations, from a forest management point-of-view. We chose a first rotation (FR) and a second rotation (SR) stand and monitored the carbon stock dynamics of these plantations over a full rotation (from the 1st to the 8th year). Our specific questions were: (i) How does successive rotation affect carbon stocks in eucalyptus plantations? (ii) What underlying factors may contribute to stock changes? Our results will provide new insights into optimal forest management practices for increasing plantation timber production as well as carbon sequestration potential.

## Materials and Methods

### Study site and plot establishment

#### Ethics statement

This research was conducted at the Dongmen Forest Farm, we confirmed that the location is not privately owned, and the sampling of plants and soils was approved by the farm. We also confirmed that the field studies did not involve endangered or protected species.

#### Study site

This study was conducted at the Dongmen Forest Farm (22°17′~22°30′N, 107°14′~108°00′ E) in Chongzuo City, of the Guangxi Zhuang Autonomous Region in China. This region is subtropical and annual rainfall averages 1300 mm, occurring primarily from April to September. Annual mean temperature is 22.3°C, with a monthly mean minimum temperature of 12.8°C and a monthly mean maximum temperature of 28.6°C. Soils are categorized as red ferrosols that are derived from sedimentary material with low to medium organic matter content and nutrient availability [26]. Soil pH ranges from 4.5~5.5 and soil depth is greater than 1 m [23]. Historically, the study area comprised a seasonal evergreen rainforest, before Chinese fir (*Cunninghamia lanceolata*), eucalyptus (*Eucalyptus* spp.) and mason pine (*Pinus massoniana*) plantations were established in the 1960s.

#### Plot establishment

Two 20-hectare eucalyptus plantations, one FR stand and one SR stand, were selected for study based on their similar climate, soil texture and topography. Soil characteristics of the two experimental sites were shown in S1 Table. The FR stand were converted directly from a 23-year-old Chinese fir plantation, while the SR stand was established after clearing a 7-year-old *E*. *exerta* plantation, which also originated from a Chinese fir plantation. After clear-cutting and prescribed burning, the sites were mechanically ploughed to a depth of 35~40 cm. The plantations were established concurrently using clonal seedlings of an eucalyptus hybrid (*E*. *urophylla* × *E*. *grandis*) in April 1998. Both plantations were established with 3.4 m × 1.7 m spacing between trees. In the first three years, the sites were fertilized with 200 kg of nitrogen, 150 kg of phosphorus and 100 kg of potassium per hectare, as well as 0.5 kg of base manure per pot. A 30-cm radius ring was weeded around each tree [23].

### Measurements

#### Overstory and understory biomass measurements

Three permanent plots (30 m×20 m each) located > 30 m apart were randomly set-up in each stand in June 1998. Each plot was further subdivided into six 10 m × 10 m subplots. Tree height and the diameter at breast height (DBH) of all woody plants in each subplot were measured every December from 1998 to 2005 (see S2 Table).

In each stand, nine sample trees with base diameters spanning from the lower to the upper end of available diameters were randomly selected outside the permanent plots, and felled for above- and below-ground biomass determination every year between 1998 and 2005. The trees were cut at a height of 5 cm above the ground. Aboveground biomass was subdivided into four components: bark, branches, leaves and stems. Similarly, the belowground portions of the sample trees were dug out and examined using the open cut method. The fresh weights of the stump roots, coarse roots (diameter>2.0 cm), medium roots (diameter 0.5~2.0 cm) and fine roots (diameter<0.5 cm) were examined separately using a balance. Five representative branches (including foliage) were sampled per tree, from the base to the crown. Tree trunks were cut into 2 m sections and segments were weighed on a balance. A disk was cut from end-to-end in each trunk segment to later determine moisture content in the laboratory. Five representative samples of each component were collected. These samples were oven-dried at 85°C to determine the moisture content. The total dry weight of each above- and below-ground component was calculated for each sample tree. The biomass of each tree component was calculated according to the allometric equations of Wen [27].

Understory biomass, including both herbs and shrubs, was destructively harvested in five randomly located 2 m × 2 m quadrats per plot. Forest floor litter was also sampled by collecting all organic material within each quadrat. All vegetation samples were oven-dried at 85°C to determine the density of dry biomass per hectare (Mg. ha^−1^).

Biomass samples were oven-dried, ground with a laboratory grinder and passed through a 1-mm sieve, before analyzing carbon concentration with a SmartChem Discrete Auto Analyzer (AMS/Westco, Italy). Carbon content was obtained by multiplying each tissue carbon concentration by the total dry weight of each component.

#### Soil sampling and analyses

Mineral soil samples were taken from depths of up to 60 cm in each stand. Soil samples were extracted from three depths (0~20 cm, 20~40 cm, and 40–60 cm) using an 8.7 cm diameter stainless steel corer. In each plot, five soil cores were collected at each depth and bulked into one composite sample. Soil samples were air dried at room temperature (25°C), passed through a 1-mm mesh sieve to remove coarse living roots and gravel, and then ground prior to chemical analysis. Meanwhile, five additional soil cores for each soil depth were sampled from each plot to measure soil bulk density; whole samples were weighed and subsamples dried at 105°C. The organic carbon content of soils was determined using a SmartChem Discrete Auto Analyzer.

### Statistical analysis

The amount of carbon stored in eucalyptus trees (TC), the understory vegetation (UC) and forest floor litter (FFC) was calculated separately, as carbon content multiplied by biomass. Soil organic carbon (SOC) stocks for each soil depth were calculated as carbon content times soil mass (soil depth × bulk density values). Total SOC values were determined by adding the SOC values of each depth together. Total ecosystem carbon (EC) was calculated as the sum of TC, UC, FFC and total SOC, and the relative contributions of each component were calculated (dividing by EC) and expressed as percentages.

Two-way analyses of variances (ANOVAs) were used to examine the differences in TC, UC, FFC, total SOC and EC between the FR and SR stands over the eight year study period. Rotation (FR or SR) and year (1 to 8 years) were treated as fixed effects. To compare SOC at each soil depth, a three-way ANOVA was conducted. Rotation, soil depth and year were treated as fixed effects. To compare carbon allocation across the ecosystem, the relative percentage of carbon in TC, UC, FFC and SOC was analyzed with two-way ANOVAs, treating rotation and year as fixed effects.

LSD multiple comparison tests were conducted to examine differences among treatments for significant interaction terms. All statistical analyses were performed with PASW version 18 and figures were drawn in Sigmaplot 11.0.

## Results

### Plant carbon stock dynamics

**Fig 1:**
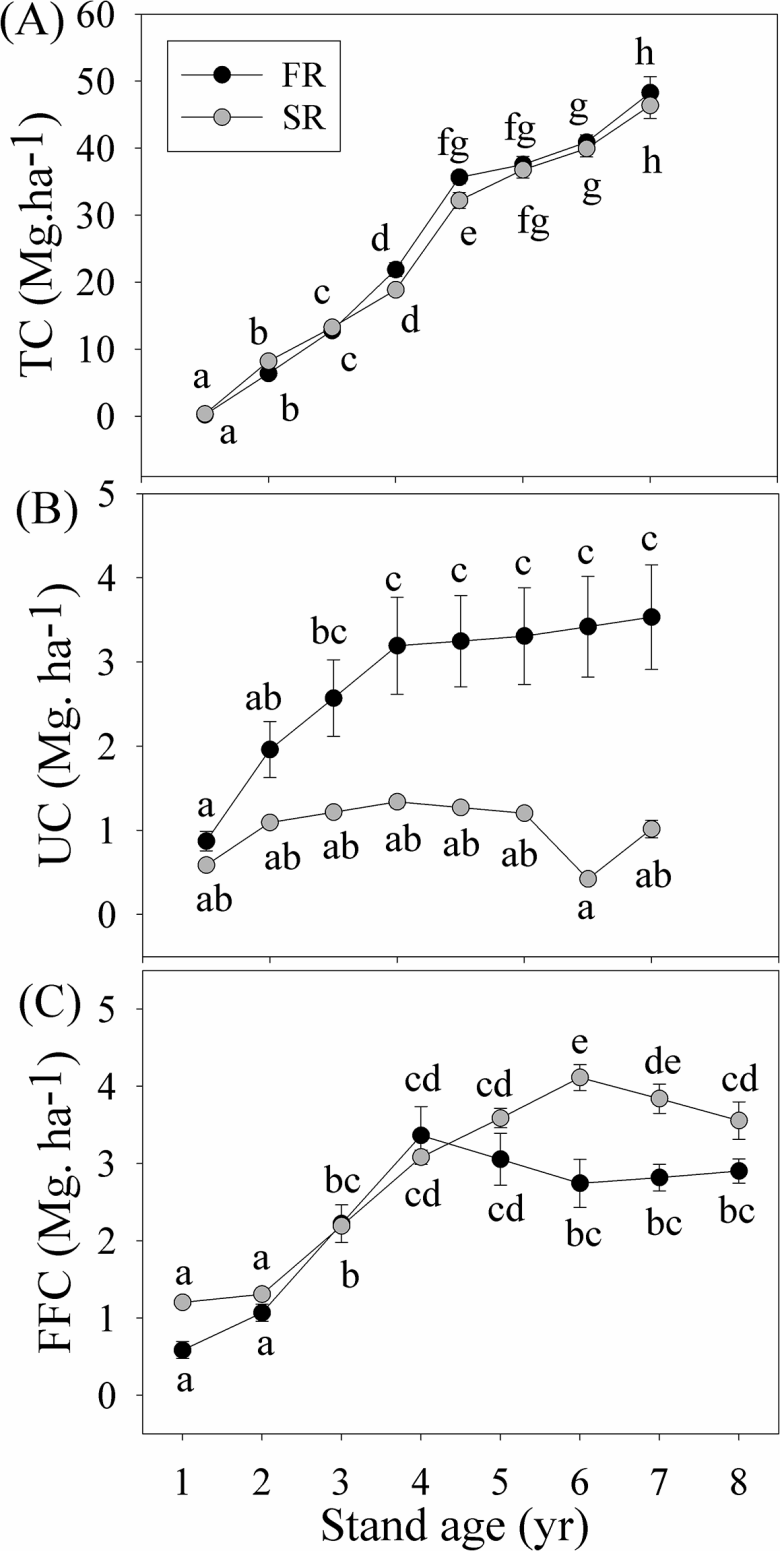
Changes in carbon stock (Mg.ha^-1^) in eucalyptus trees (TC) (A), understory vegetation (UC) (B), and forest floor litter (FFC) (C) in the first (FR) and the second rotation (SR) eucalyptus stands over a full rotation. Values with different letters were significantly different at the *p*<0.05 level.

TC stocks did not differ significantly between rotations (*p* = 0.092) (Table 1; Fig 1A). However, TC stocks of the two stands markedly increased with each year (*p*<0.001), from 0.18 Mg.ha^-1^ (FR) and 0.32 Mg.ha^-1^ (SR) in the first year, to 48.26 Mg.ha^-1^ and 46.36 Mg.ha^-1^ in the eighth year, respectively (Fig 1A). There was no interaction between the effects of rotation and year on TC stocks (*p* = 0.274) (Table 1).

**Table 1:** Results of ANOVAs analyzing the effects of rotation (first vs. second), year (1–8 years) and their interaction on the carbon stocks of eucalyptus trees (TC), understory vegetation (UC), forest floor litter (FFC), soil organic matter (SOC) and the whole ecosystem (EC) of eucalyptus plantations. Significant results (*p*<0.05) are shown in bold.

Factors	*df*	TC		UC		FFC		Total SOC		EC	
		*F*	*p*	*F*	*p*	*F*	*p*	*F*	*p*	*F*	*p*
**Rotation**	1, 32	3.018	0.092	95.761	<0.001	27.154	**<0.001**	2704.885	**<0.001**	820.266	**<0.001**
**Year**	7, 32	481.003	**<0.001**	4.406	0.002	55.008	**<0.001**	179.097	**<0.001**	1271.623	**<0.001**
**Rotation × Year**	7, 32	1.318	0.274	3.013	0.015	3.694	**0.005**	15.551	**<0.001**	11.721	**<0.001**

UC stocks were highly dependent on rotation (*p*<0.001) and year (*p* = 0.002) (Table 1). UC stocks were higher in the FR than SR stand for all study years (*p*<0.05). At the end of the rotation, UC stocks in the FR stand were 2.5 times greater than those in the SR stand (Fig 1B). Furthermore, there was a significant interaction between rotation and year effects on UC stocks (*p* = 0.015) (Table 1); UC stocks increased over time in the FR stand (from 0.87 Mg.ha^-1^ to 3.53 Mg.ha^-1^), especially over the first four years, but remained relatively constant in the SR stand (Fig 1B).

FFC stocks were greatly affected by rotation (*p*<0.001) and year (*p*<0.001) (Table 1). Mean FFC stocks were lower in the FR than the SR stand, but increased with year (Fig 1C), from 0.58 Mg.ha^-1^ (FR) and 1.20 Mg.ha^-1^ (SR) initially to 2.90 Mg.ha^-1^ and 3.56 Mg.ha^-1^, respectively, in the final study year. There was also a significant interaction between rotation and year (*p* = 0.005) (Table 1). In the FR stand, FFC stocks gradually increased over the first four years but then remained unchanged, while in the SR stand, FCC stocks increased consistently over the first six years from 1.20 Mg.ha^-1^ to 4.11 Mg.ha^-1^ (a ~3.4 times increase) (Fig 1C).

### Mineral soil carbon stock dynamics

**Fig 2:**
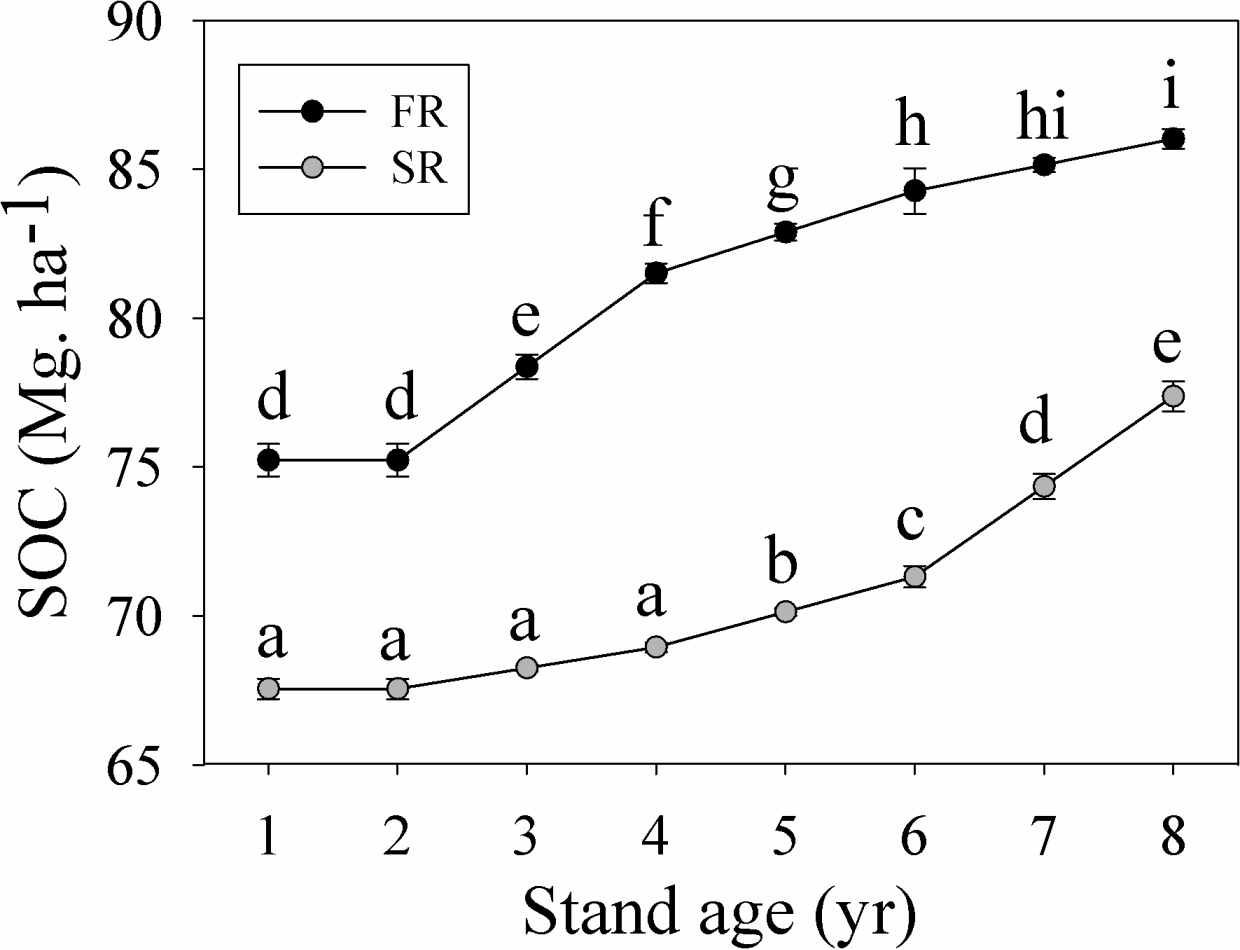
Changes in soil organic carbon (SOC) stock (Mg.ha^-1^) in the first (FR) and the second rotation (SR) eucalyptus stands. Values with different letters were significantly different at the *p*<0.05 level.

The amount of organic carbon stored in the soil differed between rotations (*p*<0.001) (Table 1), with the FR stand storing 1.2 times more SOC than the SR stand (Fig 2). In the eighth year, SOC stocks measured 86.02 Mg.ha^-1^ (FR) and 77.38 Mg.ha^-1^ (SR). SOC stocks also differed across years (*p*<0.001) (Table 1), generally increasing with stand age in both stands (Fig 2), from 75.23 Mg.ha^-1^ (FR) and 67.55 Mg.ha^-1^ (SR) in the first year, to 86.02 Mg.ha^-1^ and 77.38 Mg. ha^-1^, respectively, in the eighth year. The interaction between rotation and year was significant (*p*<0.001); while SOC stocks in the FR stand increased from the third year onwards, they only began to increase in the fifth year in the SR stand (Fig 2).

**Fig 3:**
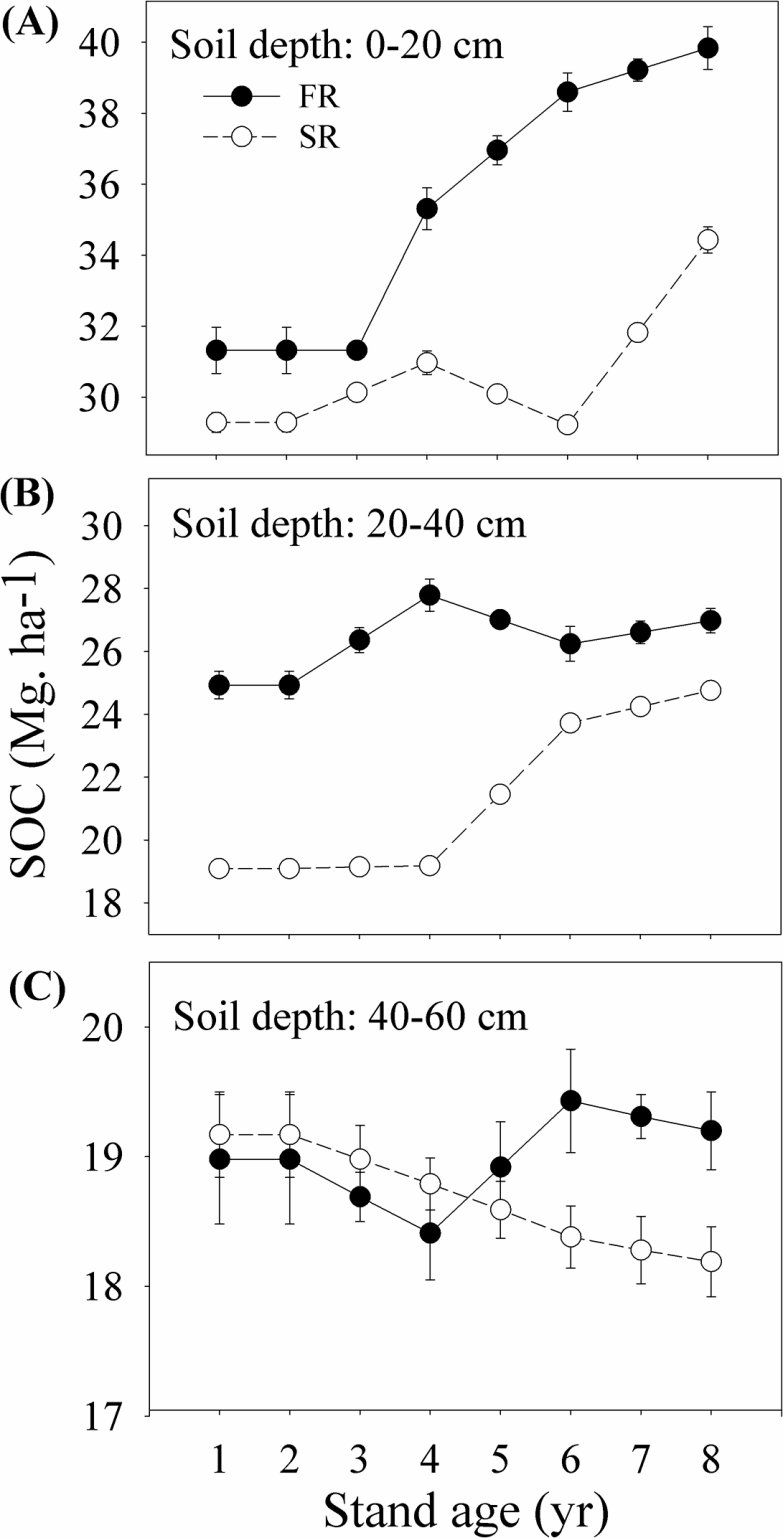
Soil organic carbon (SOC) stock (Mg.ha^-1^) at soil depths of 0~20 cm (A), 20~40 cm (B), and 40~60 cm (C) in the first (FR) and the second rotation (SR) eucalyptus stands.

Additionally, SOC stocks were highly dependent on year (*p*<0.001), rotation (*p*<0.001) and soil depth (*p*<0.001) (Table 2). Generally, SOC stock decreased with increasing soil depth and number of successive rotations, but increased with stand age (Fig 3). Topsoil (the upper 20 cm) had a higher SOC content than the other soil layers, being 1.43 and 1.83 times higher than at 20~40 cm and 40~60 cm depths, respectively. Topsoil sequestered roughly 44.49~46.31% of the total SOC by the eighth year (Fig 3A–3C). The interaction between year and soil depth was significant (*p*<0.001) (Table 2). SOC stock at 0~20 cm and 20~40 cm depths increased over time, from 30.30 Mg.ha^-1^ and 21.94 Mg.ha^-1^ in the first year to 36.97 Mg.ha^-1^ and 25.83 Mg.ha^-1^ in the eighth year, respectively, while SOC stock at 40~60 cm depths decreased over time (Fig 3A–3C).

**Table 2:** ANOVA table showing the effects of rotation (first vs. second), year (1–8 years) and their interaction on soil organic carbon (SOC) stock at 0~20 cm, 20~40 cm, and 40~60 cm soil depths. Significant results (*p*<0.05) are shown in bold.

Factors	*df*	*F*	*p*
**Year**	7, 96	157.654	**<0.001**
**Depth**	2, 96	12953.18	**<0.001**
**Rotation**	1, 96	2166.149	**<0.001**
**Year × Depth**	14, 96	53.331	**<0.001**
**Year × Rotation**	7, 96	15.191	**<0.001**
**Depth × Rotation**	2, 96	444.413	**<0.001**
**Year × Depth × Rotation**	14, 96	53.245	**<0.001**

### Whole ecosystem (EC) carbon stock dynamics

**Fig 4:**
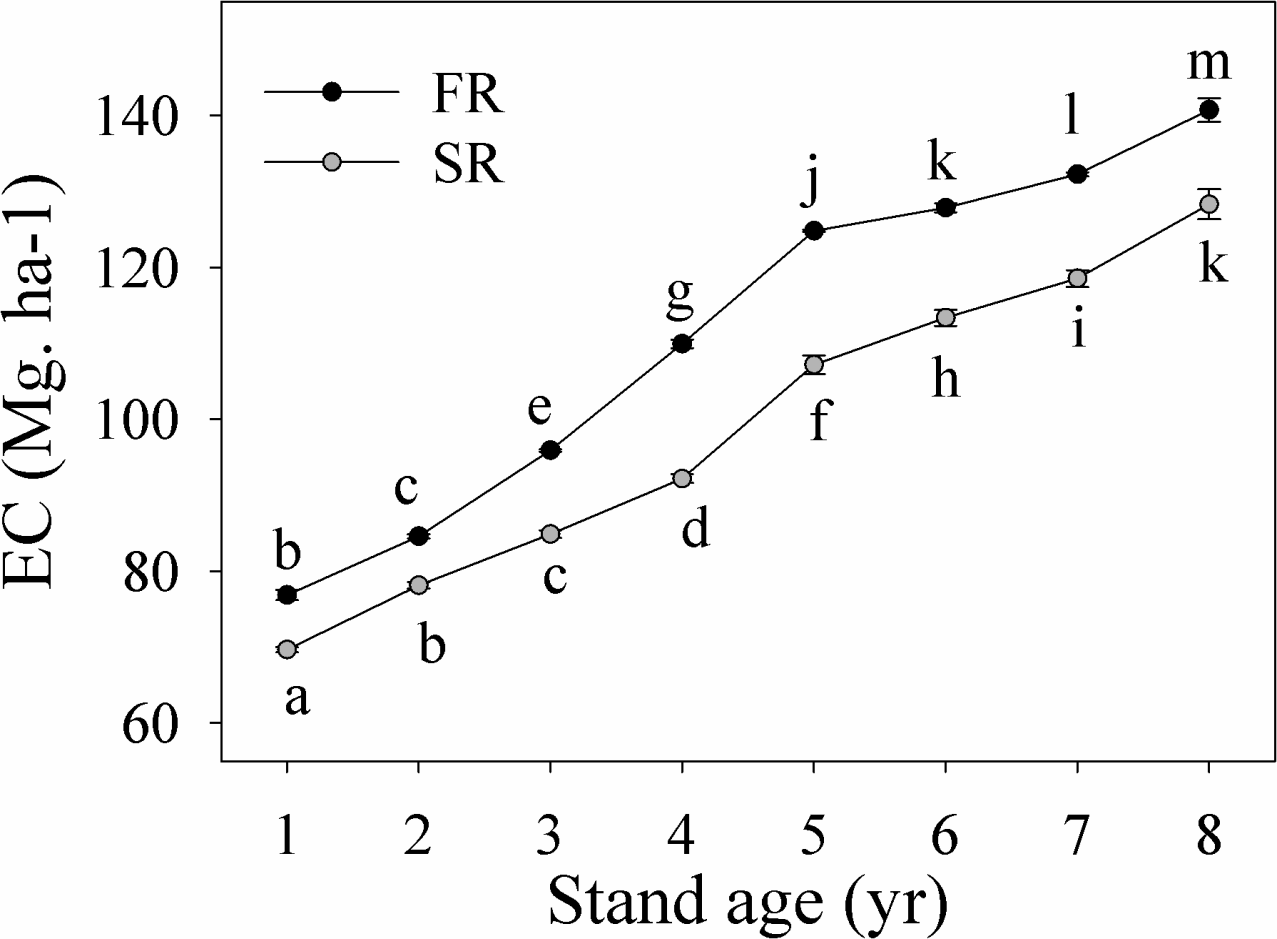
Changes in carbon stock (Mg.ha^-1^) in the whole ecosystem (EC) in successive eucalyptus plantation rotations. Values with different letters were significantly different at the *p*<0.05 level. FR: the first rotation; SR: the second rotation.

EC stocks differed between rotations (*p*<0.001) and among years (*p*<0.001) (Table 1); EC stock decreased about 1.17 times with the second successive rotation, but gradually increased with stand age over the study period (Fig 4). EC stocks increased from initial values of 76.86 Mg.ha^-1^ (FR) and 69.65 Mg.ha^-1^ (SR), to 140.72 Mg.ha^-1^ and 128.31 Mg.ha^-1^, respectively, at the end of the study (year eight). Rotation and year interacted (*p*<0.001) (Table 1): although EC stock was lower overall in the SR versus FR stand, it was similar in the two stands during the last 3 years of the study (Fig 4).

### Carbon stock allocation dynamics in the whole ecosystem

**Fig 5:**
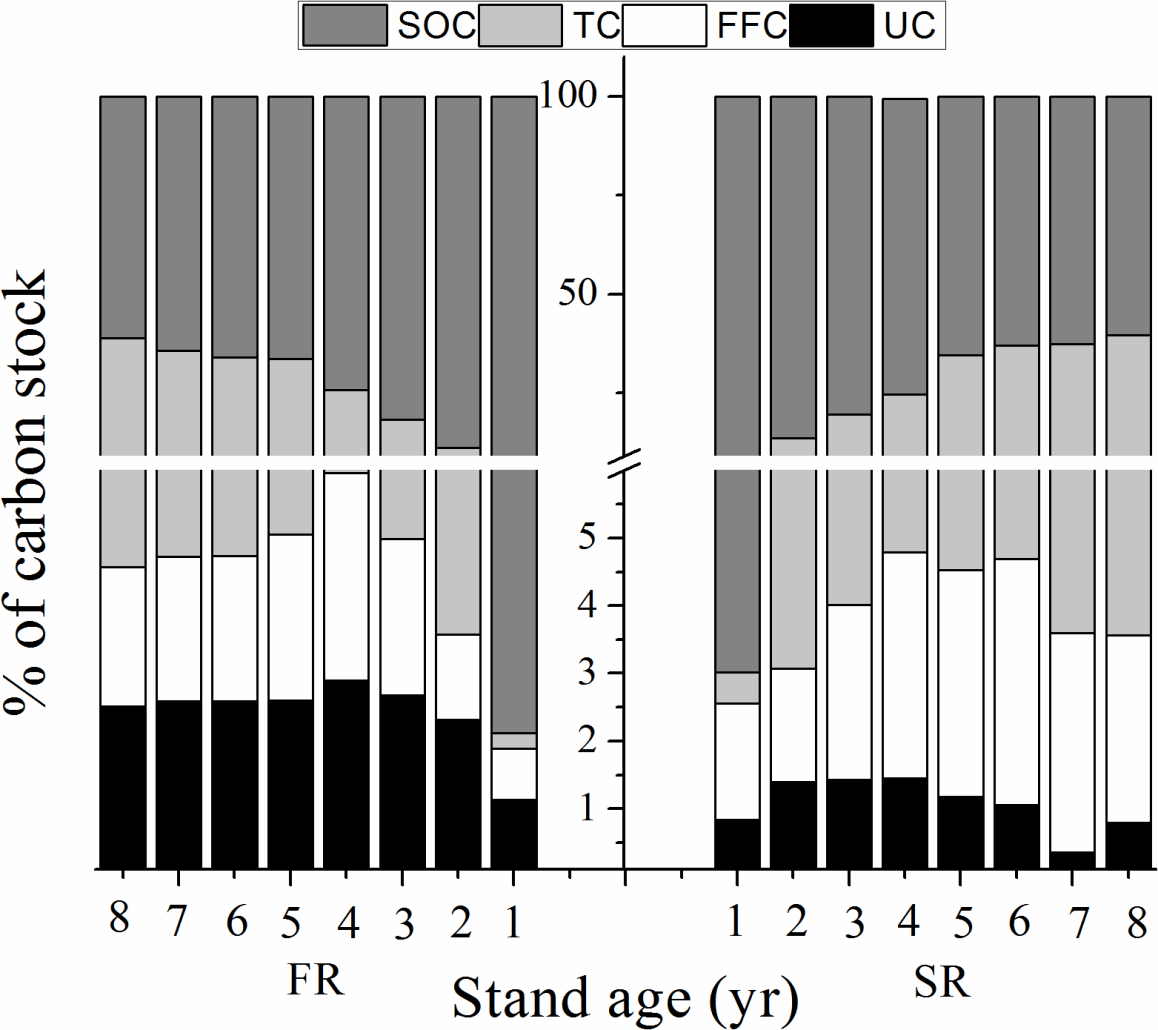
Contributions of forest components to whole ecosystem carbon stocks (expressed as percentages) in successive eucalyptus plantation rotations. TC: tree carbon stock; UC: understory vegetation carbon stock; FFC: forest floor litter carbon stock; SOC: soil organic carbon stock. FR: the first rotation; SR: the second rotation.

The relative contributions of TC (*p*<0.001), UC (*p*<0.001), FFC (*p*<0.001) and SOC (*p*<0.001) stocks to the whole ecosystem carbon pool differed between the rotations (Table 3). SOC and UC stocks comprised a greater percentage in the first versus second successive rotation, at 75.06% versus 73.77% (SOC) and at 2.42% versus 1.05% (UC). In contrast, the contribution of TC and FFC stocks was higher in the second rotation, increasing from 20.50% to 22.39% (TC) and from 2.02% to 2.79% (FFC). The relative contributions of each forest component to carbon stock were ranked as follows: SOC > TC > UC > FFC in the FR stand, and SOC > TC > FFC > UC in the SR stand (Fig 5).

**Table 3:** Results of ANOVAs analyzing the effects of rotation (first vs. second), year (1–8 years) and their interaction on the relative contributions of eucalyptus trees (TC), understory vegetation (UC), forest floor litter (FFC), and soil organic matter (SOC) to the whole ecosystem carbon pool. Significant results (*p*<0.05) are shown in bold.

Factors	*df*	TC		UC		FFC		SOC	
		*F*	*p*	*F*	*p*	*F*	*p*	*F*	*p*
**Rotation**	1, 32	28.276	**<0.001**	77.443	**<0.001**	85.899	**<0.001**	20.083	**<0.001**
**Year**	7, 32	624.661	**<0.001**	2.742	**0.024**	35.776	**<0.001**	1055.377	**<0.001**
**Rotation × Year**	7, 32	1.151	0.357	1.721	0.139	3.387	**0.008**	1.949	0.094

The relative contributions of TC (*p*<0.001), UC (*p* = 0.024), FFC (*p*<0.001) and SOC (*p*<0.001) stocks to whole ecosystem carbon stock varied significantly over time (Table 3). The proportion of TC content increased, while SOC content decreased over time; the proportions of UC and FFC contents initially increased the first four years before gradually decreasing the following years (Fig 5). There was a significant interaction between the effects of rotation and year on the proportion of FFC (*p* = 0.008) (Table 3): FFC stocks in the FR stand increased consistently over the first four study years only, while in the SR stand, FCC stocks increased over the whole experimental period (Fig 5).

## Discussion

### Successive rotations had no effect on TC stocks

Our results clearly demonstrate that forest TC content was not significantly affected by successive rotations, only declining very slightly in the second rotation (Fig 1A). Many previous studies have found that successive rotations have negative effects on stand biomass and the TC content of plantations [20, 28]. For example, Zhang et al. [20] reported that stand biomass of Chinese fir plantation was reduced by 24% from the first to the second rotation, and by 40% from the second to the third rotation. However, Subedi et al. [29] reported that the SR pine plantations outperformed the FR, understory vegetation and forest floor from the FR were considered as important nutrient sources of pines in the SR.

An increase in soil bulk density was considered a major factor in the observed decrease in biomass and carbon stock potential of Chinese fir plantations over successive rotations [20]. Other studies have reported a close association between soil bulk density and SOC levels [20, 30, 31]. Higher soil bulk density may inhibit plant root growth and nutrient uptake via alteration of the micro-conditions of the soil [20, 32]. Previous studies have reported that soil bulk density decreased [33] or increased [34] below plantations compared with natural forests. In our study, the initial soil bulk density in the SR stands was lower than the FR stand (as shown by S1 Table). Wen [27] showed that although soil nutrients such as Ca and Mg decreased over successive rotations in eucalyptus plantations, the soil bulk density also decreased about 12%. Site preparation practices for plantations (e.g. removal or burning of residue, tillage, weed control, and fertilization) can greatly influence soil bulk density and tree growth [35, 36]. Some experiments have shown that the aboveground biomass production of eucalyptus plantations over a full rotation can be up to twice as high in plots with large amounts of harvest residue at planting compared to plots where the forest floor layer has been removed [37]. This lower soil bulk density may have been one of the most important contributors to the maintenance of high tree biomass in the second rotation.

Understory competition is another factor affecting the growth of many young forest plantations [38]. Understory plant communities in managed plantations are often considered as competitors for water, nutrients, and light resources [39]. Strong understory competition represented one of the primary causes for lower growth rates of pine [40]. However, previous studies have observed that understory vegetation growth has been shown to be greatly reduced over successive rotations in eucalyptus plantations [23, 25]. As the canopy closes, understory vegetation receives limited light and consequently grows very slowly. Less competition from understory vegetation can result in higher biomass and growth rates of eucalyptus trees [27]. Although soil degradation may occur over the course of successive rotations, as reported by Fialho and Zinn [41] and Qi et al. [22], weakened competition from understory plants and lowered soil bulk density values can still allow for consistent growth and biomass accumulation in eucalyptus trees, as seen here in the SR stand.

### UC stocks decreased over successive rotations

Our results suggest that successive rotation has significantly negative effects on carbon stock in understory vegetation (Fig 1B). This finding is consistent with that of previous studies that also showed a decline in the biomass and carbon stock potential of understory vegetation with an increasing number of successive rotations [20, 23, 24]. Wen [27] also found a decline in species diversity over successive rotations in eucalyptus stands.

Understory vegetation growth in plantations can be influenced by many factors such as climate, management practices, soil and stand type [12, 42, 43]. The biomass of understory vegetation has also been shown to decrease with increasing disturbance [19]. Anthropogenic disturbances such as fertilization, prescribed burning, site preparation, tree cutting and weeding may greatly affect the composition and growth of understory vegetation [17, 18, 44–47]. These management practices can also negatively influence the availability of soil nutrients [37, 44, 47]. Newmaster et al. [19] found that repetitive prescribed burning and mechanical site preparation resulted in a decline in the biomass of understory vegetation. In our study, only one round of disturbance occurred in the FR stands. In contrast, the SR stand experienced more frequent disturbances. More disturbance may contribute to a decline in UC stocks in eucalyptus plantations with multiple rotations.

### FFC stocks increased over successive rotations

The litter on the forest floor plays an important role in the regulation of nutrient cycling and the maintenance of soil fertility in forest [37, 48, 49]. FFC stocks depend on many factors, such as forest species composition, litter type and litterfall mass, the decomposition rate and the soil micro-environment [10, 19, 48]. Litter production in eucalyptus plantations was found to increase about 16.2% in the SR successive rotation [27]. Yang et al. [35] found that soil polyphenol oxidase activity and phenol content changed substantially with continuous planting of eucalyptus rotations. These findings were supported by similar results from other studies where greater litter production occurred over successive rotations [50, 51].

FFC stocks are affected by soil conditions such as soil temperature, moisture and fertility [19]. Residues can maintain the soil at a lower temperature and a higher humidity [52]. Harvest and site preparation practices, such as slash harvesting and/or extensive removal of residue, may change soil microclimate and soil nutrient supply [36], greatly affecting the production and decomposition of floor litter and detritus [48]. Soil nutrient availability can also greatly affect the activities of decomposers and microbes [53]. An increase in litterfall mass accompanied by a reduction of the decomposition rate results in the accumulation of litter over successive rotations (Fig 1C). Tree species composition was also considered as an important factor controlling the litter decomposition rate [48], increased in litter production over successive rotation may be partly explained by changes in understory species compositions as reported by Wen [27].

### SOC stocks decreased over successive rotations

The soil carbon pool is determined by the balance between carbon input and the release of carbon during decomposition [12, 16]. SOC stocks highly depend on forest type, soil structural and chemical properties, and the silvicultural strategy [12, 16, 54]. Many earlier studies have demonstrated that SOC content is significantly affected by a plantation’s successive rotation regime [41, 55]. Litter input into the soil both throughout and between rotations can have a major impact on soil properties and soil organic matter content [38]. Decrease in SOC stocks in the SR stand may be partly caused by accumulation of forest floor litter during successive rotations.

The initial soil conditions prior to new plantation establishment may greatly affect later tree growth, species compositions and litter production [29, 37]. Differences in soil properties (such as nutrient, physical structure, microenvironment, etc.) at the beginning of plantation establishment can affect SOC stock dynamics of plantations [49, 54]. There is also an increasing concern in soil nutrient deficiency and soil degradation in short-rotation plantations [41, 55, 56]. We found that soil total N, total P, hydrolysable-N, available-P, available-K, exchangeable-Ca, exchangeable-Mg in the SR stand were all lower than the FR stand (see S1 Table), indicating fertility depletion occurred over successive rotations. Although SOC content can be gradually recovered after the establishment of plantations (as shown by Fig 3), the recovery is limited as SOC will again be lost during preparations for the next successive rotation. For example, SOC stock had declined 12 years after establishing *E*. *grandis* plantations in Australia [57]; in contrast, SOC stocks had fully recovered after 14 years in an *E*. *camaldulensis* plantation on a Quartzipsamment [58].

The observed decline in SOC content in the SR eucalyptus stand in our study was more likely to be attribute to poor site preparation and current short-rotation forestry strategies [43]. Sites of eucalyptus plantations are typically prepared by slash harvesting and burning all litter, residues and understory vegetation; the soil is then ploughed before planting. Removal of harvest residues can result in a great loss in the available nutrients such as cations [36]. Although burning of residue materials can immobilizes nitrogen and reduce the losses of nitrogen [36, 49], it also results in the losses of carbon and some nutrients in volatile forms [36]. Furthermore, after intensive residue removal and tillage, the surface soil are hence easily subject to erosion during the rainy season, especially before the stand canopy is closed [54]. These are probably the most important causes of the decline in SOC stocks over successive rotations [9, 47].

### Changes in the relative contributions of TC, UC, FFC, and SOC to the total carbon stocks

The assessment of total ecosystem carbon stocks requires consideration of the stability of different carbon pools, such as TC, UC, FFC and SOC stocks. In our study, overstory trees and mineral soils were two dominant carbon pools in eucalyptus stands; however, as UC stocks decreased and FFC stocks increased over successive rotations, the relative contribution of FFC stock grew in the SR stand. Carbon within the litter layer is considered to be less stable than carbon in mineral soils [28]. Nave et al. [59] found that losses in FFC stocks caused by harvesting were much higher than those of SOC stocks. Litter accumulation combined with a loss of understory vegetation led forest floor litter to sequester more carbon over successive rotations. In contrast to reports from Korean pine plantations, where aboveground tree biomass was the major contributor to total ecosystem carbon sequestration [60], mineral soils were the dominant carbon pool in eucalyptus plantations. Significant reductions in SOC and UC stocks resulted in lower overall EC content in the SR stand. Zhang et al. [20] also observed a decline in carbon stocks in successive rotations of Chinese fir plantations. Further studies are needed to explore possible factors contributing to such declines in yield and carbon stock in plantations over successive rotations.

## Conclusions

Our eight-year experiment found that although consistent eucalyptus tree biomass production was maintained over two successive rotations, UC and SOC stocks significantly decreased (note FFC stocks increased). The lower UC and SOC stocks resulted in lower overall EC stock over successive rotations. Mineral soils and overstory trees were the two dominant carbon pools in the eucalyptus plantations; the relative contribution of FFC stocks to whole ecosystem carbon increased and that of UC stocks decreased over a full rotation. These changes were attributed to current successive rotation regimes of eucalyptus plantations including residue removal/burning, tillage, fertilization and weed control practices, etc. Our results suggest that for sustainable development of eucalyptus plantations, current short rotation regimes need to be altered in order to strike a balance between yield and carbon stock.

## Supporting Information

S1 Table Soil characteristics of the experimental sites at Dongmen Forest Farm. (DOC)

S2 Table Mean ± SE values of stem density, diameter at breast height (DBH), and height of eucalypt tree in the first and the second rotation (FR and SR) stand over a full rotation (from the 1st to the 8st year). The differences between rotations were not statistically significant (*p*>0.05), excepting for the first 2 years.
(DOC)
