# Supplementary material for: Effects of Successive Rotation Regimes on Carbon Stocks in Eucalyptus Plantations in Subtropical China Measured over a Full Rotation
Source: PLoS One. 2015 Jul 17;10(7):e0132858. doi: 10.1371/journal.pone.0132858 (PMC4505904; doi:10.1371/journal.pone.0132858)
Supplement: S1 Table — (DOC) [file pone.0132858.s001.doc]

| **Site** | **Depth**  **(cm)** | **Bulk density**  **(g. cm^-3^)** | **Organic matter**  **(g. kg^-1^)** | **pH**  **(H_2_O)** | **Total N**  **(g. kg^-1^)** | **Total P**  **(g. kg^-1^)** | **Total K**  **(g. kg^-1^)** | **Hydroly-**  **sable-N**  **(mg. kg^-1^)** | **Available-P**  **(mg. kg^-1^)** | **Available-K**  **(mg. kg^-1^)** | **Exchan-**  **geable-Ca**  **(mg. kg^-1^)** | **Exchan-**  **geable-Mg**  **(mg. kg^-1^)** |
| --- | --- | --- | --- | --- | --- | --- | --- | --- | --- | --- | --- | --- |
| **^a^ FR** | 0-20 | 1.14 | 24.21 | 4.70 | 0.85 | 0.67 | 2.35 | 119.88 | 2.32 | 40.32 | 127.22 | 56.28 |
|  | 20-40 | 1.42 | 14.86 | 4.80 | 0.65 | 0.72 | 2.11 | 83.26 | 1.16 | 23.12 | 71.04 | 35.47 |
|  | 40-60 | 1.43 | 11.30 | 5.10 | 0.42 | 0.60 | 2.37 | 42.70 | 0.48 | 15.05 | 49.71 | 13.11 |
| **^b^ SR** | 0-20 | 1.09 | 23.31 | 4.49 | 0.79 | 0.52 | 2.65 | 101.1 | 0.70 | 37.53 | 84.75 | 30.92 |
|  | 20-40 | 1.19 | 17.65 | 4.61 | 0.65 | 0.49 | 2.68 | 51.01 | 0.16 | 18.32 | 43.89 | 17.69 |
|  | 40-60 | 1.23 | 11.29 | 5.01 | 0.52 | 0.43 | 2.52 | 29.15 | 0.05 | 13.05 | 24.86 | 8.33 |

^a^ FR: the first rotation; ^b^ SR: the second rotation.
