# Supplementary material for: Effects of Successive Rotation Regimes on Carbon Stocks in Eucalyptus Plantations in Subtropical China Measured over a Full Rotation
Source: PLoS One. 2015 Jul 17;10(7):e0132858. doi: 10.1371/journal.pone.0132858 (PMC4505904; doi:10.1371/journal.pone.0132858)
Supplement: S2 Table — The differences between rotations were not statistically significant (p>0.05), excepting for the first 2 years. (DOC) [file pone.0132858.s002.doc]

| **Stand age**  **(Years)** | **FR** | | | **SR** | | |
| --- | --- | --- | --- | --- | --- | --- |
|  | **Stem density**  **(ha^-2^)** | **DBH**  **(cm)** | **Height**  **( m)** | **Stem density**  **(ha^-2^)** | **DBH**  **(cm)** | Height  (m) |
| **1** | 1683 ±25.3 | 1.90 ±0.10 | 2.80 ±0.10 | 1683 ±58.5 | 2.37 ±0.03 | 3.23 ±0.07 |
| **2** | 1683 ±25.3 | 6.43 ±0.15 | 8.70 ±0.15 | 1672 ±65.6 | 6.80 ±0.10 | 10.13±0.26 |
| **3** | 1683 ±25.3 | 7.80 ±0.15 | 11.70 ±0.06 | 1672 ±65.6 | 7.90 ±0.15 | 11.93 ±0.22 |
| **4** | 1683 ±25.3 | 9.17 ±0.18 | 14.30 ±0.45 | 1644 ±45.4 | 8.60 ±0.15 | 14.37 ±0.24 |
| **5** | 1645 ±14.7 | 11.03±0.15 | 16.23 ±0.15 | 1611 ±58.0 | 10.73 ±0.27 | 15.87 ±0.20 |
| **6** | 1600 ±16.7 | 11.17±0.12 | 17.07 ±0.22 | 1600 ±57.7 | 10.90 ±0.25 | 17.57 ±0.20 |
| **7** | 1600 ±16.7 | 11.33±0.13 | 17.83 ±0.15 | 1600 ±57.7 | 11.10 ±0.25 | 18.20 ±0.20 |
| **8** | 1594 ±19.9 | 12.10±0.17 | 18.27 ±0.33 | 1567 ±42.1 | 11.93 ±0.27 | 18.40 ±0.20 |
